# The Tobacco Habit among the Young

**Published:** 1897-11

**Authors:** 


### THE TOBACCO HABIT AMONG THE YOUNG.

Whatever may be thought concerning the effect of tobacco-smoking on the adult, the opinion that it is deleterious in the extreme on the young is unanimous and decided. Of late years juvenile smoking has been spreading like an epidemic in all countries of the world, and is attacking both the physical and moral health of nations. In France, in Germany, and in this country, efforts have been made to check its further inroads. In some parts of Germany, as also in portions of the United States, laws have been enacted prohibiting persons under the age of eighteen from smoking, and rendering it a punishable offense for any one to give or sell tobacco to children. In France numerous societies have been formed for the suppression of the vice. In no country has this habit increased with the young to a greater extent than in England. The advent of the cheap cigarette is doubtless chiefly responsible for this state of affairs. To see boys of seven or eight years old puffing their cigarettes is quite a common occurrence in London, and particularly in this the case in the East End. However, when a packet containing five cigarettes can be bought for two cents, the fact that smoking has become so general can scarcely be wondered at. Sir William Harcourt, in his last speech on the Budget, referred to the large increase of revenue received from tobacco, in these words: "I believe it is mainly due to the great increase in the consumption of cigarettes, which are especially attractive to our youthful population." He added: "I am told of one manufacturer who makes two million cigarettes a day who hardly made any a few years ago." It has been proposed in Great Britain, as a remedy for the evil, that the members of the medical profession should make a move in the matter, and urge on the managers of schools the importance of special teaching exposing the harmfulness of juvenile smoking, and should also make such representations to Parliament and the Government as might lead to efficient legislation. It is difficult to see in what manner this vice can be checked among children unless by repressive measures. If the medical profession in this country were to exert themselves with a similar object in view the habit might be yet stopped. There is no question in the case of interference with a person's liberty, it is simply a matter of health and morals. In Paris some months ago, at a meeting of the Society against the abuse of tobacco, it was decided to submit a petition to the Chamber of Deputies praying that "all telegraph messengers and school-boys should be prevented from smoking, and also that tobacconists should be forbidden to sell their wares to mere infants whose lips should know no other pleasure than the cheeks of their mother." Though the wording of this petition is rather curious, it is quite to the point. There is a humorous side to the question of children smoking. In a book on tobacco lately published is the following paragraph: "It was the custom in England about the middle of the seventeenth century, for children going to school to carry in their satchel with their books a pipe of tobacco, which their mothers took care to fill early in the morning, it serving them in place of breakfast. At the accustomed hour every one laid aside his book to light his pipe, the master smoking with them and teaching them how to hold their pipes and draw in the tobacco." At the present day Dutch children smoke pipes, and little boys of five and seven years old calmly discuss these calumets of peace as they proceed to school. Still this does not alter the fact that smoking injures the health of the young. The excessive use of tobacco is harmful to many adults; it tends to deteriorate the moral character in the same way as the inordinate use of chloral or bromide of potassium will deprave the mind, by lowering the tone of certain of the nerve centers. If this is so with grown men, how much more forcibly must the case apply to immature youths?—*Pediatrics.*

---

At the fourth annual meeting of the American Medical Publishers' Association, held in St. George Hall, Philadelphia, on May 31st, the following resolution was introduced and adopted:

"Whereas, the Imperial Granum Company has announced the withdrawal of all its advertising patronage from the Lay Press, and signified its intention of using Medical Mediums only in the future, therefore be it

"*Resolved*, That the American Medical Publishers' Association, in session at Philadelphia, hereby indorses and commends this action of the Imperial Granum Company, and further recommends this course to other manufacturers who desire the support and co-operation of the medical profession."

